# Surfaces environmental monitoring of SARS-CoV-2: Loop mediated isothermal amplification (LAMP) and droplet digital PCR (ddPCR) in comparison with standard Reverse-Transcription quantitative polymerase chain reaction (RT-qPCR) techniques

**DOI:** 10.1371/journal.pone.0317228

**Published:** 2025-02-03

**Authors:** Simona Spiteri, Irene Salamon, Luna Girolamini, Maria Rosaria Pascale, Federica Marino, Carlo Derelitto, Laura Caligaris, Simone Paghera, Manuela Ferracin, Sandra Cristino

**Affiliations:** 1 Department of Biological, Geological, and Environmental Sciences, University of Bologna, Bologna, Italy; 2 Department of Medical and Surgical Sciences, University of Bologna, Bologna, Italy; 3 IRCCS Azienda Ospedaliero-Universitaria di Bologna, Bologna, Italy; 4 Copan Italia, Brescia, Italy; Fondazione Don Carlo Gnocchi, ITALY

## Abstract

The persistence of Severe Acute Respiratory Syndrome Coronavirus 2 (SARS-CoV-2) on substrates, and the impact of fomites on Coronavirus Disease 19 (COVID-19) transmission, is until now, widely discussed. Consequently, further investigations are required for a correct risk assessment in high-risk facilities such as hospitals, healthcare facilities (HCFs), and long-term care facilities (LTCFs). Therefore, appropriate surveillance and disinfection programs represent the best approach to guarantee the safety of these communities. This study proposes an environmental SARS-CoV-2 surfaces routine monitoring approach in HCF and communities’ settings, to provide rapid and effective evaluation of surface hygienic conditions and the effectiveness of applied sanitization measures. Surfaces samples (n = 118) were collected using the SRK^®^ kit (Copan Italia) from 2020 to 2023. Three molecular techniques were compared: Reverse Transcription Loop mediated isothermal AMPlification (RT-LAMP, Enbiotech), Reverse-Transcription quantitative polymerase chain reaction (RT-qPCR) (RT-qPCR, Seegene) and droplet digital PCR (ddPCR, Bio-Rad). For ddPCR, two RNA extraction methods were compared: TRIzol LS (Invitrogen) versus QIAmp Viral Mini kit (QIAGEN), showing how the latter is more suitable for surfaces. Regarding the quantitative ddPCR results, the ROC analysis allowed to reduce the manufacturer cut-off for droplets number (from 3 to 1) for the positive samples. Moreover, a new cut-off for the viral RNA copies’ number/μL for each target (N1 and N2) on environmental monitoring was fixed at 2,82. The results obtained using the QIAmp kit, suggested that the N2 target is more stable in the environment and could be most suitable for the virus environmental detection. The percentage of positive samples was similar among the techniques (26% for RT-LAMP, 36% for ddPCR and 23% for RT-qPCR). Using RT-qPCR as reference method, a sensitivity (SE) of 30% for RT-LAMP and 41% for ddPCR was observed. By contrast, specificity (SP) was higher for RT-LAMP (75%) respect to ddPCR (66%). Comparing the faster RT-LAMP with the sensitive ddPCR the 26% and 74% of SE and SP for RT-LAMP, were reported. The low sensitivity for RT-LAMP and ddPCR could be explained with the use of clinical rather than environmental kits, other than the changing in the virus prevalence during the sampling campaign. Although the RT-LAMP requires improvements in term of SE and SP, this research presents an innovative environmental monitoring and prevention method for SARS-CoV-2, that could be extended to other pathogens that are under environmental surveillance.

## Introduction

Severe Acute Respiratory Syndrome Coronavirus 2 (SARS-CoV-2) is the etiological agent of a severe acute respiratory syndrome. It belongs to the large respiratory viruses’ family of *Coronaviridae*, so-called because of the spike protein, which is shaped like a crown. Clinical manifestations of SARS-CoV-2 infection, known as COVID-19 (Corona Virus Disease), include flu-like symptoms such as fever, cough, dyspnea and gastrointestinal complaints that can evolve to systemic inflammation and multiorgan dysfunction [1].

SARS-CoV-2 outbreak in China at the end of December 2019 has rapidly turned into a pandemic. On 31 December 2019, Chinese health authorities notified an outbreak of pneumonia cases of unknown etiology in Wuhan City, Hubei, China. The initial cases were associated to an exposure to the Seafood and wildlife city market, where mammalian species were on sale before the outbreak. For this reason, the involvement of living animals in the transmission chain was hypothesized. Even if genomic comparison shows that the viruses most closely related to SARS-CoV-2 originate from bats (96% similarity), the exact role of bats on SARS-CoV-2 zoonotic origin remains still uncertain [2].

Starting from the pathogen’s isolation in January 2020, the World Health organization (WHO) declares pandemic status on 11 March 2020 [3]. One year since the first reported cases of pneumonia in China, on 31 December 2020, WHO issued its first emergency use validation for a COVID-19 vaccine [4]. Since then, research efforts have focused on the development and refinement of vaccines and drugs against COVID-19 [5]. At the same time, several pandemic waves and unpredictable new variants of SARS-CoV-2 have occurred, affecting 775,583,309 people and causing 7,050,691 deaths until 2 June 2024 [6].

Transmission occurs mainly through the air: SARS-CoV-2 is carried by an infected patient to a healthy one through droplets produced by breathing, talking or coughing. In this way, the virus infects the upper respiratory tract binding the human Angiotensin-Converting Enzyme 2 receptor (ACE2), expressed in nasal and oral tissues, through the envelope Spike protein [1]. However, ACE2 receptors are also present in other human tissues such as urogenital, cardiovascular and gastrointestinal tissues [7,8]. In fact, at the beginning of this pandemic, gastrointestinal symptoms in COVID-19 patients suggested an involvement of ACE2 receptors in the orofecal transmission of SARS-CoV-2, too [9]. This question has been solved by several studies in recent years. For example, real-time reverse transcription–polymerase chain reaction (RT-qPCR) detects the presence of SARS-CoV-2 RNA in fecal samples in hospitalized COVID-19 patients [10–13].

From a general perspective, the main routes of transmission of respiratory viruses include direct person-to-person contact via airborne droplets or aerosols and indirect contact via fomites or hands of infected people. Furthermore, a possible route of transmission could involve resuspended viruses from fomites [14]. Nevertheless, there are currently differing opinions on the role of fomites in the transmission of SARS-CoV-2: some authors have demonstrated a low risk of contamination, while others reported a higher risk [15]. On the other hand, it has been proven that SARS-CoV-2 persists on inanimate surfaces such as other nosocomial pathogens. Hydrophobic and non-porous surfaces (e.g. plastic, metal, glass, ceramics and rubber surfaces) keep it in stable conditions at room temperature preserving its infectivity for several days depending on the type of surface involved, for example on metal or plastic surfaces it can persist for up to 3–4 days) [16]. A recent systematic review on SARS-CoV-2 cultures for assessing fomite transmission, reported as replication-competent SARS-CoV-2 can be readily detected on fomites in specific settings. Moreover, replication-competent SARS-CoV-2 may be found in fomites in relation to the timing of sampling from symptom onset, severity of the patients’ illness, and the RT-qPCR C_t_ < 30 (high viral titer) [17–23].

Past and the current public health emergencies show how countries are unprepared for the occurrence of future pandemics and that focusing entirely on clinical and diagnostic research of emerging diseases has proven lacking. The One Health approach, an interdisciplinary approach that considers humans and the environment in which they live as one system, represents the key point to understanding the spread of emerging diseases and their impact on human health and the environment. Therefore, an integrated collaboration between clinical research and environmental surveillance for emerging pathogens is the key to preserving people’s well-being before getting the disease [24,25]. Considering all this, environmental monitoring of SARS-CoV-2 can improve knowledge of pandemic events and help to overcome it.

The monitoring of surfaces to assess the persistence of SARS-CoV-2 in healthcare facilities (HCF) such as long-term facilities and hospitals in presence of patience or after the sanitization treatment, represents a preliminary step to face an emergency and to control the spread of nosocomial infections. Moreover, it represents a best approach to verify the effectiveness of sanitization procedures avoiding ineffective sanitization treatment, that in a short- and long-term lead to the accumulation of disinfectants residues on the environment, increasing the resistance among pathogenic microorganisms. A routine screening of community environments such as schools, work facilities and leisure centers, therefore, could be introduced and undertaken as preventive measures.

However, the study of surfaces contamination using the common molecular procedures has its limitations. For example, monitoring an entire building by analyzing surface swabs with RT-qPCR, is disadvantageous because the assay is expensive, time-consuming, requires sophisticated equipment and qualified staff. Because of this, innovative and rapid methods are required.

Loop mediated isothermal AMPlification (LAMP) was developed in year 2000 to overcome the limits of RT-qPCR, and it was primarily used for diagnosis and detection of diseases [26]. It is a qualitative amplification system, characterized by an isothermal profile setting (about 60–65°C), to sidestep the temperature changes required by conventional polymerase chain reaction (PCR). This makes LAMP a faster method than RT-qPCR and conventional PCR. Moreover, unlike the RT-qPCR and PCR, amplification and detection of nucleic acid are carried out in a single step using four or six specifically designed primers and a polymerase with strand displacement activities. Due to this process, massive amplification is possible, as DNA can be amplified up to 10^9^ times in one hour [27,28]. In these years of pandemic, DNA-LAMP protocols have been adapted to perform RNA amplification. Unlike LAMP for DNA, Reverse Transcription LAMP (RT-LAMP) involves the use of reverse transcriptase to convert RNA into complementary DNA (cDNA) that is used for amplification. This system allows the diagnosis of a multitude of viral infections [29] and has been implemented for SARS-CoV-2 diagnosis [30–32]. Nevertheless, RT-LAMP use is still limited to clinical applications. Instead, with few exceptions [33], the use of RT-qPCR predominates for the environmental detection of SARS-CoV-2 [34–37]. However, RT-qPCR returns false positive (FPR) and false negative (FNR) results when quantifying less abundant targets and relies on a standard curve to quantify targets [38].

The 3rd generation PCR, the droplet digital PCR (ddPCR), is the latest generation of PCR. It has been developed to overcome the challenges of PCR and as RT-qPCR and RT-LAMP, ddPCR has been used widely for SARS-CoV-2 diagnostics [39–41] and sporadically for environmental surveillance [42–44]. ddPCR works by partitioning samples into thousand to millions of droplets which may contain the target copies. Dividing the sample into several portions ensures that amplification proceeds simultaneously, and not in a single bulk reaction as RT-qPCR. In addition, ddPCR is also more inhibition-resistant than RT-qPCR, as observed for clinical samples, including faces, and environmental samples, including soil, water and wastewater and it increases practical sensitivity when detecting low target copy numbers in other nucleic acids presence [38,45]. Then, once amplification is completed, the ddPCR platform provides absolute quantification based on Poisson statistics [46] rather than a calibration curve [38]. This provides greater repeatability and accuracy in quantification and, compared to RT-qPCR in many applications, ddPCR has reached a higher analytical sensitivity for the detection of various target molecules in environmental and clinical samples [45]. Considering this, comparing these molecular biology techniques aims to evaluate the performance of innovative detection methods, to understand their strengths and limitations and to define which method best suits for the routine environmental monitoring of SARS-CoV-2.

The aim of this study is to perform the SARS-CoV-2 surveillance on several types of surfaces, to test the contamination levels in hospital and community environments. Since there are no adequate guidelines, this study proposes the assessment of innovative techniques such as ddPCR and RT-LAMP for the environmental detection of SARS-CoV-2 by comparing them to the RT-qPCR, the clinical gold standard for the detection of the pathogen. Because of the limited availability of kits for surfaces matrices when the study began, clinical reagents were used. Therefore, the purposes of the study included the effectiveness of adapting clinical protocols to environmental matrices SARS-CoV-2 routine monitoring, with a focus on the evaluation of two different nucleic acid extraction methods: a chemically driven method, the TRIzol LS Reagent (TRI), and a spin column-based method, the QIAmp Viral Mini kit (QIA), and three molecular techniques characterized by different level of complexity as RT-LAMP, RT-qPCR and ddPCR. The final goal is to provide a rapid and sensitive approach to better test the effectiveness of sanitization procedures undertaken during ordinary and extraordinary routine environmental surveillance, that could be adopted also for other nosocomial and emerging pathogens.

## Materials and methods

### Sample collection

A total of 118 surface samples were collected in the city of Bologna, northern Italy, from positive patients houses and healthcare facilities. Among them, special attention was paid to long-term care facilities for the increased susceptibility of hosts and private hospitals where patients were treated for complications of COVID-19 or directly acquired it as nosocomial infection. The sampling campaign started in January 2022, and it was carried out until May 2023. Several types of surfaces were chosen, including mobile phones, tableware, TV remote controls, magazines, toothbrushes, door handles, sink faucets, toilet seats, toilet drains, bed rails and hospital supplies used by COVID-19 patients such as medicine packs, emergency bedside controls, lunch tables and walking aids. According to the WHO Guideline [47], on regular surfaces sampling was carried out using 25 cm^2^ templates, while for small objects or irregular surface such as door handles, faucets, etc., the entire surface was sampled. The SRK^®^ kit (Copan Italia, Brescia) provided with a FLOQSwabs^®^ with regular tip and placed in sterile collection tubes containing 3 ml of SRK medium were used. After sampling, FLOQSwabs^®^ eluted in SRK medium were inactivated in the thermal bath at 50 ± 5 °C for 30 minutes and then stored at -80°C until use [48].

### Viral RNA extraction by TRIzol Reagent and QIAmp Viral Mini kit

The first phase of the study focused on the ddPCR analysis performed using two nucleic acid extraction systems commonly used in clinical settings were compared: i) an organic extraction protocol using TRIzol LS Reagent (Invitrogen, Waltham, Massachusetts, US) and ii) the column-based QIAmp Viral Mini kit (QIAGEN, Hilden, Germany) according to manufacturer’s instructions. TRI is a monophasic solution of phenol and guanidine isothiocyanate designed to isolate nucleic acids from tissues of human, animal, plant, yeast, or bacterial origin. QIA provides RNA purification from plasma, serum and other cell-free body fluids. Both methods were performed following the manufacturer’s instructions. After the extractions, the RNA recovery was 60 μL for QIA method and 40 μL for TRI extraction. The RNA was stored at − 80 °C until ddPCR analysis.

### ddPCR assay

The ddPCR was performed using the Bio-Rad SARS-CoV-2 ddPCR Kit (Bio-Rad Hercules, California, US) targeting human RPP30 gene, SARS-CoV-2 N1 and N2 genes. RT-qPCR primers and probes panel provided by kit, were developed according to Centers for Disease Control and Prevention (CDC) guidelines [49]. The Bio-Rad QX200 Droplet Digital PCR Systems (Bio-Rad) was used following the manufacturer instructions. In detail, a volume of 5.5 μL of template was added to the master mix composed of 1.1 μL 2019-nCoV CDC ddPCR Triplex Assay, 2.2 μL reverse transcriptase, 5.5 μL Supermix, 1.1 μL 100 mM DTT and 6.6 μL of nuclease-free water to obtain a total volume of 22 μL. Then, 20 μL of reaction mix added with the template underwent droplet generation by the QX200 droplet generator (Bio-Rad). Droplet-partitioned samples were then transferred to a 96-well plate, sealed and amplified to thermal cycler according to the following thermal profile: 50°C for 60 min, 95°C for 10 min followed by 40 cycles of initial PCR activation at 94°C for 30 seconds and 55°C for 30 seconds, then of 98°C for 10 min, 4°C for 30 minutes. A particular focus was placed on the number of droplets generated during the analysis to intercept a potential matrix effect: when the analysis did not return a droplet number greater than or equal to 10.000 and if the number of positive droplets was less than three, ddPCR analysis was repeated. QuantaSoft Software (QuantaSoft 1.7.4.0917, Bio-Rad) and QX Manager Software (QX Manager 1.2.345 Standard Edition, Bio-Rad) were used to collect and analyze the dataset obtained by ddPCR reaction. According to manufacturer’s instructions for use, samples were considered positive when the QuantaSoft software showed a number of positive droplets equal to, or greater than three for at least one of the two viral detected targets. Each ddPCR dataset obtained from QX Manager Software was filtered and reported on an Excel file through an in-house Python script (Python 3.11.0) to avoid transcription errors.

### RT-qPCR

RT-qPCR was carried out after the ddPCR analysis. The design of the RT-qPCR analysis was influenced by the results of the ddPCR. In fact, considering the ddPCR results discussed further below, and with the aim of optimizing the work, only samples purified using the QIA extraction kit were analyzed with RT-qPCR.

RT-qPCR was carried out using the Allplex SARS-CoV-2/Flu A/Flu B/RSV Assay (Seegene, Seoul, South Korea), a clinical multiplex RT- PCR assay designed to detect N gene, RdRP gene and S genes for SARS-CoV-2, influenza A, influenza B and respiratory syncytial virus (RSV). The total volume of reaction was 20 μL containing 10 μL of RNA template. Reaction conditions were 50°C for 20 min, then 95°C for 15 min followed by 3 cycles of initial PCR activation at 95°C for 10 seconds, 60°C for 40 seconds, and 72°C for 20 seconds, followed by 42 amplification cycles of 95°C for 10 seconds, 60°C for 15 seconds and 72°C for 10 seconds. The results of analysis were interpreted as positive if one or more genes were detected with a Cycle threshold (Ct) value ≤ 40 [44].

### RT-LAMP: RNA extraction and analysis

SARS-CoV-2 RNA extraction and detection for RT-LAMP were carried out through the environmental SARS-CoV-2 kit (Enbiotech S.r.l., Palermo, Italy) targeting SARS-CoV-2 S gene using the IC-Gene Plus platform (IC-Gene, Angri, Italy). The RNA was extracted from the swab transport medium using the extraction buffer provided by the kit, following a ratio of 1:5: briefly, 40 μL of SRK medium were transferred into a new tube containing 160 μL of extraction buffer provided by kit. After 2 minutes of incubation at room temperature, 20 μL of RNA extract was added into previously prepared Primer Mix tubes provided by the manufacturer containing 20 μL of LAMP Mix and 30 μL of mineral oil. Finally, each tube was loaded into the IC-Gene Plus platform to start the analysis process. A positive result for the RT-LAMP was determined by a visible sigmoid curve for each sample consultable on a tablet provided by the IC-Gene app and by the “+” sign on the screen (IC-Gene app version 3.5.6-indium). Moreover, positive and negative control provided by the kit was performed.

### Statistical analysis

The results obtained were analyzed in terms of positive and negative samples reported for each technique. An initial comparison between the extraction methods, QIA *versus* TRI, was made focusing on the number of droplets generated to perform the ddPCR analysis. The software R (version 4.1.1) was used with the Shapiro-Wilk test [50] to evaluate the normal distribution of data, subsequently when the normality of distribution was not observed the Wilcoxon signed-rank test [51] was used. The significance was fixed using the *p* value (*p*) at <0.05. Moreover, using the GraphPad Prism software (GraphPad 10.0.0) a receiver operating characteristic (ROC) analysis was performed to assess the accuracy of ddPCR [52]. Moreover, the cut-off for ddPCR results, was established to discriminate the positivity of a sample, in relation to the droplets generated and the number of RNA copies detected in relation to the extraction method adopted. Then, considering that the gold standard for SARS-CoV-2 in clinical filed is represented by RT-qPCR, it was retained as the reference method and its results were compared to RT-LAMP and ddPCR. Finally, a comparison between ddPCR and RT-LAMP was performed considering the most sensitive ddPCR as the reference method. Therefore, among detection techniques, were calculated:

- The percentage of positive and negative samples for each technique;
- The concordance using the standardized Cohen’s coefficient (k) to measure the level of real agreement between two qualitative measurements [53] (in this case, the statistical units are positive and negative samples);
- The sensitivity (SE), specificity (SP) and 95% confidence interval (CI);
- The positive (PPV) and negative predictive values (NPV).

## Results

### Analysis of extraction methods on ddPCR results

The results obtained showed that a high number of positive samples were detected by ddPCR when using the QIA extraction method, with 42 out of 118 samples (36%) testing positive, compared to 13 out of 118 samples (11%) using the TRI extraction method.

Considering that the Bio-Rad ddPCR data analysis using the manufacturer’s software requires at least 10,000 droplets to perform the Poisson statistical analysis, an in-depth analysis was conducted on the number of droplets generated by the two extraction methods. The number of droplets obtained using TRI was fewer than 10,000, in 34% (40/118) of samples, in the range of 0–9889 (minimum-maximum). To increase the number of droplets produced, the samples were reprocessed twice: first by repeating the extraction protocol, and second by extending the ethanol evaporation time from 10 to 25 minutes. In the first case the number of droplets remains low, by contrast the improvement of protocol permits to recover all 40 samples, obtained an increase on number of generated droplets (all samples ≥ 10,000). Regarding the QIA method, only the 11% (13/118) of the samples initially showed a low number of droplets, ranging from 5,926 to 9,946 (minimum-maximum). However, after reprocessing these samples without any changes to the protocol, an increase in droplet’s number was observed, resulting in 100% of the samples reaching the correct number of droplets.  

To estimate these differences, an in-depth analysis regarding the number of droplets obtained using the two extraction methods was performed, before and after the change of evaporation time and the link with origin of surfaces was performed.

Since the small sample size, it was important to determine the distribution to select the most appropriate statistical method. Therefore, the Shapiro-Wilk test was performed before and after reprocessing the samples. Before modifying the TRI protocol, the Shapiro-Wilk test for the TRI extraction method dataset showed a significant departure from normality (W = 0.93572, *p-*value = 2.595^−5^). In contrast, the Shapiro-Wilk test confirms a normal distribution for the QIA dataset (W = 0.98442, *p*-value = 0.1909). Therefore, since the TRI dataset presents a non-normal distribution, the Wilcoxon signed-rank test was used to identify any statistically significant differences between the two extraction methods. From the Wilcoxon signed-rank test returned statistically significant difference between the two groups (W = 5322.5, *p*-value = 0.001775) before the sample reprocessing. After the reprocessing, TRI and QIA datasets present a non-normal distribution (TRI: W = 0.90783, *p*-value = 6.057^−7^, QIA: W = 0.96106, *p*-value = 0.001721). In contrast to the previous case, the Wilcoxon signed-rank test revealed no statistically significant differences (W = 7048.5, *p*-value = 0.8697, W = 6875.5, *p*-value = 0.8697).

Assuming that the droplet generation issue was related not only to the extraction method used but also to the type of surface analyzed, particularly its origin (private homes or HCFs), further analysis was carried out. This assumption arises from the observation that surface sanitation is less rigorous in domestic setting compared to HCFs and some detergent ingredients may interfere with the extraction method, affecting droplet generation. Our data displayed how the samples with number of droplets < 10000 was detected in 8 samples using both extraction methods. In detail, 1 comes from private homes and 7 from HCFs. Considering each extraction method, TRI remains the methods with low number of droplets, mainly found in the HCF environments (11 versus 29 samples, respectively). The same trend, respecting the origin of samples, was reported from QIA (1 private home versus 12 HCF). From the Wilcoxon signed-rank test, it was found that for the TRI extraction method, there were no statistically significant differences in droplet generation between the two settings, home and HCF, before (W = 1850.5, *p*-value = 0.09921) and after the samples’ reprocessing (W = 1651, *p*-value = 0.6069) with extended ethanol evaporation time. Likewise, no statistically significant differences were found for the QIA extraction method between the two environments, before (W = 1688, *p*-value = 0.4685) and after (W = 1495, *p*-value = 0.7138) reprocessing of the samples.

Because of droplets generation problems using the TRI extraction method and the lower number of positive samples obtained, the subsequent phase of the study focused on the RT-qPCR analysis, was performed using RNA extracted with the QIA method, aiming to optimize resources.

### Evaluation of SARS-COV-2 gene target and cut-off value for ddPCR detection

Considering the quantitative ddPCR results, to assess the suitability of clinical extraction protocols such as TRI and QIA for environmental matrices like surfaces, both the number of positive droplets and the copy number for each target were considered. A detailed evaluation of ddPCR data revealed that the most frequently detected target was N2 alone or in combination with N1 for QIA protocol (Table 1).

**Table 1:** Samples positivity by target and extraction method using ddPCR.

Samples positivity by SARS-CoV-2 target		
Detected target	TRI positive samples No. (%)	QIA positive samples No. (%)
**N1**	8 (62%)	4 (10%)
**N2**	3 (23%)	15 (36%)
**N1 + N2**	2 (15%)	23 (55%)
**Total**	13	42

Moreover, the receiver operating characteristic (ROC) analysis revealed the cut-offs regarding the number of droplets and of the RNA copies’ number of positive samples as shown in Table 2.

**Table 2:** Cut-off for droplets and copies number for each target of SARS-CoV-2 analyzed by ddPCR.

Extraction method	Cut-off			
	No. of droplets		No. of copies/μL	
	N1	N2	N1	N2
**TRI**	1.5	3.5	32,430	43.72
**QIA**	1.5	1	9.6	2.82

**Fig 1:**
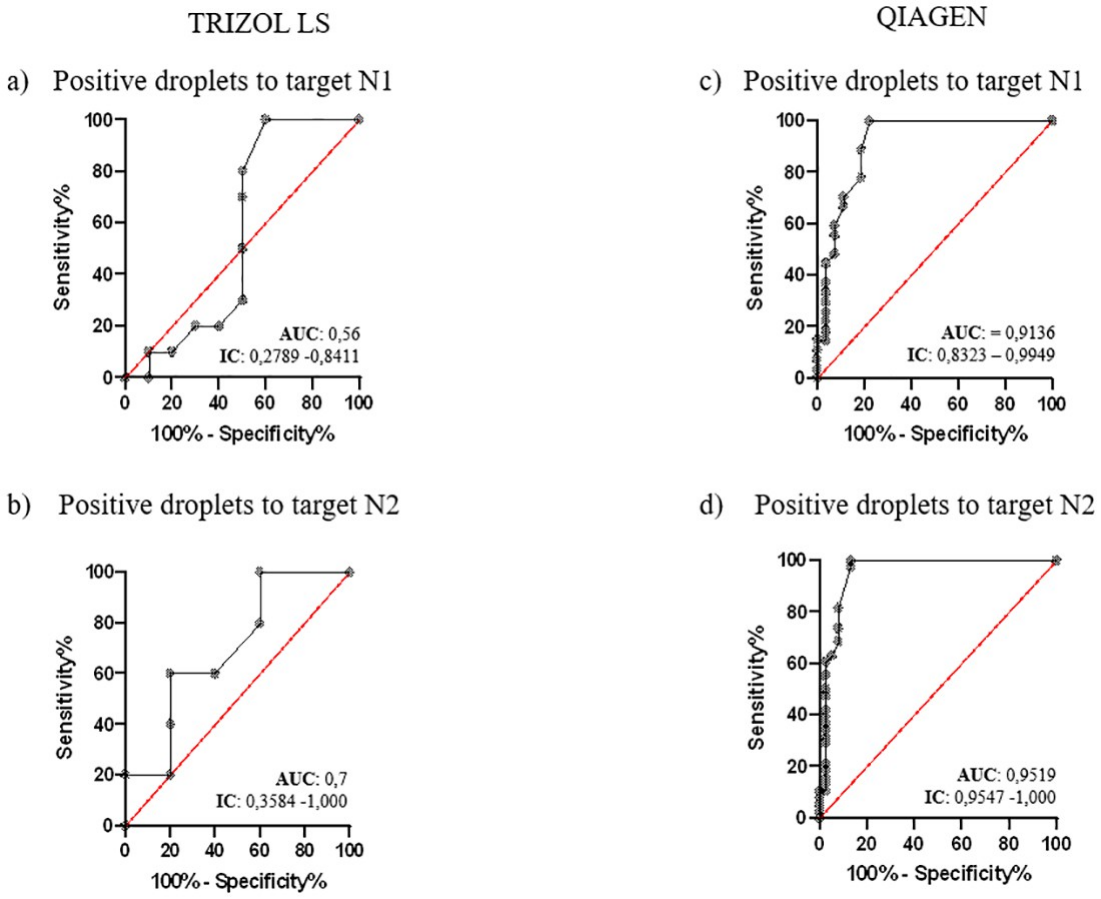
The ROC curves assess the accuracy of both extraction protocols through the number of droplets obtained during the partitioning phase of ddPCR for each target (N1 and N2). a) and b) using TRI method; c) and d) using QIA.

**Fig 2:**
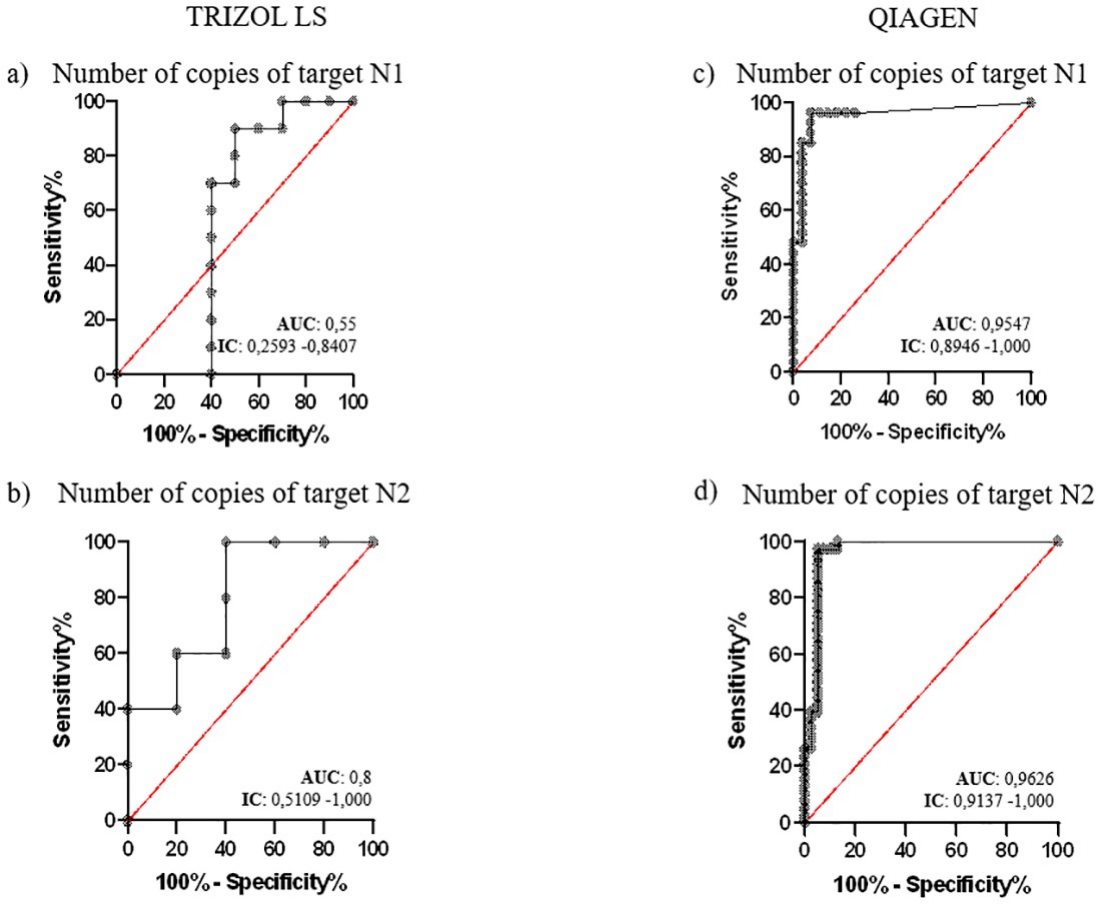
The ROC curves were used to assess the accuracy of both extraction protocols through the number of copies of N1 and N2 obtained after the droplets’ reading process (N1 and N2). a) and b) using TRI method; c) and d) using QIA.

In addition, using the ROC analysis, the ROC curves and the respective area under curve (AUC), concerning the cut-off of droplets’ number (Fig 1) and the RNA copies’ number (Fig 2), respectively was elaborated.

Observing the trend of the ROC curves for both targets, N1 (Fig 1A and 1B) and target N2 (Fig 1C and 1D), higher AUC values were observed for the QIA extraction method (N1 = 0.9136, N2 = 0.951) respect to the TRI extraction (N1 = 0.56; N2 = 0.7). Therefore, QIA extraction ddPCR presented accuracy levels of 91.4% and 95.1% for N1 and N2 targets, respectively. On the contrary, TRI extraction ddPCR presents accuracy values of 56.0% and 70.0% for N1 and N2.

To investigate the contamination levels on surfaces using a clinical kit, the droplet number cut-offs were investigated through ROC analysis. For the QIA extraction method, cutoffs of 1.5 droplets for target N1 and 1.0 droplets for target N2 were determined. When using the TRI extraction method, cut-offs of 1.5 droplets for N1 and 3.5 droplets for N2 were reported.

The ROC statistical analysis shown in Fig 2 highlights the differences between the two extraction methods. Observing at the trend of the ROC curves for target N1 (Fig 2A and 2B) and target N2 (Fig 2C and 2D), the AUC takes on a larger value for the QIA extraction method (N1 = 0.9574, N2 = 0.9626) than for the TRI extraction (N1 = 0.55; N2 = 0.8), demonstrating a higher accuracy of QIA (N1: 95.7%, N2: 96.2%) compared to TRI (N1: 55.0%, N2: 80.0%). As well as the number of droplets, the cut-off for total RNA copy number per surface sample analyzed was 32,430 copies for target N1 and 43.72 copies for target N2 when using the TRI extraction method. These values decrease significantly whit the QIA extraction method, with cut-offs of 9.665 copies for target N1 and 2.820 copies for target N2.

### Comparison among the ddPCR, RT-LAMP and RT-qPCR

The comparison was performed using both extraction methods for ddPCR, with “gold-standard” RT-qPCR as reference method. Table 3 displays the trend of positive samples obtained among the technique.

**Table 3:** Positive SARS-CoV-2 surface samples for each analytical method.

Samples	RT-qPCR No. (%)	RT-LAMP No. (%)	ddPCR No. (%)	
			QIA	TRI
**Positive**	27/118 (23%)	31/118 (26%)	42/118 (36%)	13/118 (11%)
**Negative**	91/118 (77%)	87/110 (74%)	76/118 (64%)	105/118 (89%)

A high number of positive samples were obtained by ddPCR, followed by RT-LAMP and RT-qPCR. Interesting results were found regarding the RT-LAMP that are in line with RT-qPCR. The concordance among the techniques was calculated by using Cohen’s Kappa (k) measure of agreement as described in Table 4.

**Table 4:** Concordance and Cohen’s Kappa (k) measure of agreement among techniques.

Compared techniques	Concordance between techniques			k Value
	Positive concordant samples (True positives) No.	Negative concordant samples (True negatives) No.	Total concordance No. (%)	
**RT-qPCR** **vs ddPCR** **(QIA RNA extraction)**	11	60	71 (60%)	0.1 **(Slight agreement)**
**RT-qPCR** **vs ddPCR** **(TRI RNA extraction)**	2	80	82 (69%)	-0.1 **(No agreement)**
**RT-qPCR vs** **RT-LAMP**	8	68	76 (64%)	0.0414 **(Slight agreement)**
**ddPCR** **(QIA RNA extraction)** **vs RT-LAMP**	11	56	67 (57%)	0.01 **(No agreement)**
**ddPCR** **(TRI RNA extraction) vs RT-LAMP**	1	75	76 (64%)	-0.130 **(No agreement)**

Considering RT-qPCR as reference methods, ddPCR performed with QIA showed a lower percentage of concordance results respect to TRI (60% versus 69%). This concordance was assessed using Cohen’s K statistics, which measure reliability. Cohen’s k for QIA was 0.1, indicating slight agreement according to Cohen’s Kappa interpretation scale, while the TRI method showed no agreement, with a Cohen’s k of -0.1.

However, this level of observed agreement should be evaluated in the context of the previously described droplet generation problems and the higher number of negative samples obtained by ddPCR after TRI extraction compared to QIA (105 and 91, respectively), which could introduce a bias. This assumption is confirmed by no agreement returned by Cohen’s k scale (k = -0.1).

A comparison was made between the qualitative RT-LAMP and the reference method RT-qPCR. RT-LAMP detected a high number of positive samples compared to RT-qPCR (31 versus 27). In this case, the two methods showed a concordance of 64% with Cohen’s k of 0.04, that confirms a slight agreement between the methods. Finally, RT-LAMP was compared to ddPCR, considering ddPCR as the reference method. Once again, an important role in these differences is played by the nature of the extraction methods. RT-LAMP detected fewer positive surfaces compared to QIA extraction method but more than the TRI method. The analysis of concordance showed higher concordance between RT-LAMP and TRI compared to QIA (64% versus 57%). However, this was contradicted by Cohen’s k, which showed a lower value for TRI than for QIA (-0.130 and 0.01, respectively), indicating no agreement.

Table 5 shows different value of SE and SP obtained comparing RT-qPCR with ddPCR, depending on the extraction method. A higher SE was obtained for the QIA extraction (QIA: 41% vs. TRI: 7%). In contrast, a higher SP for the extraction in TRI is reported (QIA: 66% vs TRI: 88%).

**Table 5:** Comparison between detection techniques. Sensitivity (SE), Specificity (SP), Positive (PPV) and Negative Predictive Values (NPV).

Compared techniques	SE (%)	SP (%)	VPP (%)	VPN (%)
**RT-qPCR** **vs** **ddPCR** **(QIA RNA extraction)**	41 (95% CI: 0.40–0.42)	66 (95% CI: 0.65–0.67)	26	79
**RT-qPCR vs** **ddPCR** **(TRI RNA extraction)**	7 (95% CI: 0.06–0.08)	88 (95% CI: 0.87–0.89)	15	76
**RT-qPCR vs** **RT-LAMP**	30 (95% CI: 0.29–0.31)	75 (95% CI: 0.74–0.76)	26	78
**ddPCR** **(QIA RNA extraction)** **vs** **RT-LAMP**	26 (95% CI: 0.25–0.27)	74 (95% CI:0.73–0.75)	35	64
**ddPCR** **(TRI RNA extraction)** **vs** **RT-LAMP**	8 (95% CI: 0.07–0.09)	71 (95% CI:0.70–0.72)	3	86

Moreover, the comparison between RT-qPCR and RT-LAMP shows an SE value of 30% and an SP of 75%.

When comparing QIA extraction ddPCR and RT-LAMP, the values of SE and of SP are 26% and 74% respectively. By contrast, considering TRI extraction for ddPCR as a reference method, the values obtained are 8% and 71% respectively.

## Discussion

Understanding the hygienic conditions of community settings is important for public health, as it allows the identification and mitigation of environmental pathogen spread, thereby preventing nosocomial and communities’ new infections. Therefore, regularly updated guidelines for environmental monitoring of SARS-CoV-2 and appropriate corrective actions (e.g., sampling methods, identification techniques, surface sanitization procedures etc.) are urgently needed, not only for the COVID-19 disease which the world is slowly learning to live with, but also for any future pandemics. During the design phase of this study, the choice of the most suitable swab for sampling was carefully considered. According to the WHO Guideline for surface sampling of SARS-CoV-2 [47], in many previous studies, the classical rayon swabs have been used [54–56]. Our choice differs from the WHO guidance for several reasons. Primarily, PCR requirements influenced this decision: calcium alginate and cotton swabs are not recommended for PCR analysis because they may contain substances that inactivate some viruses and inhibit molecular tests [57]. In addition, the properties of the FLOQSwabs^®^ and SRK^®^ transport medium played a significant role in our selection. In fact, FLOQSwabs^®^, offer a customizable sampling solution to suit the research’s requirements such as the size of the tip and the distance to the breaking point on the shaft, making them compatible with several tube sizes. Its flocked tip allows a capillarity-driven sample uptake and an efficient elution of the biological specimen due to the absence of a disorganized fiber structure trapping the sample. Moreover, SRK^®^ medium neutralizes anti-microbial cleansing agents on surfaces, providing a clear picture of the pathogen present [58].

Concerning the choice of each technique, a premise must be made. No kits for environmental matrices such as surfaces were commercially available when the study began. Therefore, considering the lack of knowledge regarding the number of particles of SARS-CoV-2 able to spread the infection, a high sensitivity technique like ddPCR was chosen for its proven ability to detect low target concentrations, such as those of a virus outside its natural host.

The first phase of study, started during the pandemic period comparing two extraction methods as QIA (columns methods) and TRI (chemical methods), to observe the impact on sample partitioning and positive samples detected. A recent study on nucleic acid extraction methods for SARS-CoV-2 proves the sturdiness of QIA in several different conditions, but it could be an expensive method if used in analytical routine [59]. Therefore, comparing these two different extraction methods aimed to assess which method is more suitable for surfaces considering the economic impact.

The results obtained confirm the best performance of QIA extraction respect to TRI, considering the number of positive samples obtained and the number of droplets obtained.

Generally, ddPCR analysis requires a generation of a great number of droplets, as having many amplification events allows the application of Poisson statistics, which are essential to ddPCR. This statistical distribution is also known as the ’distribution of rare events’ because it could be applied when the probability of success is very small, the number of tests is very high and the product of the probability of success and the number of tests is constant [46]. Therefore, an accurate Poisson analysis can be performed by optimizing the ratio of the number of positive events (positive droplets) to the total number of independent events (the total number of droplets). The number of at least 10,000 generated droplets was chosen by the manufacturer as the conventional cut-off point for the applicability of the Poisson statistics. According to this standard, the number of droplets obtained using the TRI method was fewer than 10,000 for 40 samples. Thus, 34% of the samples (40/118) were initially not eligible for the Poisson statistics.

The reprocessing of samples using the same extraction methods confirmed the presence of some interference on droplets production using TRI method. We hypothesized that incomplete evaporation of the ethanol used to wash RNA during the final steps of TRI extraction could interfere with the droplets’ generation during the ddPCR sample preparation. Extending the ethanol evaporation time from 10 to 25 minutes, compared to the original protocol, resolved this issue. The same phenomenon was observed in rare cases for the QIA extraction method, with 11% (13/118) of samples initially producing fewer than 10,000 droplets. However, repeating the ddPCR analysis resulted in an increase to more than 10,000 droplets. Probably, we suppose that mostly manipulated surfaces contain more contaminants, that would affect the downstream application of TRI extraction, considering also the type of extraction methods based on two different approaches: a chemically driven method (TRI) versus a spin column base method (QIA). TRI is a monophasic solution of phenol and guanidine isothiocyanate designed to isolate nucleic acids and proteins of human, animal, bacterial and yeast origin. In contrast, QIA is a spin column method designed to purify viral RNA from plasma, serum and other cell-free body fluids. It has been documented in the literature that the use of extraction methods such as phenol extraction (on which TRI extraction is based) or silica adsorption using guanidinium and potassium salts can alter nucleic acid partitioning during extraction. Indeed, both phenol and silica have weakly dissociated hydroxyl groups. Guanidinium cations can form hydrogen bonds between the negatively charged phosphate groups of nucleic acids and the hydroxyl groups of the phenol or silica, causing nucleic acids to become trapped in the liquid phase of the phenol or onto the solid silica phase [60]. From this perspective, this study suggests that certain matrix components may interfere with one or more solvents used during the RNA purification process. In contrast, a spin column method such as QIA could retain better substances affecting droplets’ generations during ddPCR, preserving RNA. Moreover, the highest number of positive samples in ddPCR was obtained using the user-friendly QIA RNA extraction protocol. This kit not only requires minimal manipulation by the operator, but it seems to be less affected by factors such as the operator and matrix type.

Furthermore, the analysis of samples with a low number of droplets also considered their origin. Many samples came from hospitals, which are often treated with commercial disinfectants, in contrast to private homes. The high number of samples with low number of droplets, were found in hospital using both extraction methods, whereas this phenomenon was less detected in private homes. The Wilcoxon signed-rank test showed no statistically significant differences in droplets generation between home and HCF, before and after the extension of the ethanol evaporation time. It is inferred that the origin of the sample, HCF or home, does not affect the generation of droplets. Rather, these results support the thesis that the extraction method has a greater impact on the generation of droplets. However, these data should be confirmed with a larger sample set.

The analysis of ddPCR results, considering the targets present in the panel, shows a large number of samples positive for target N2 with N1 when the QIA extraction method was used. The results obtained with ddPCR using QIA extraction method suggest that this portion of the nucleocapsid phosphoprotein gene could persist better on surfaces. This hypothesis is supported by the results of the ROC analysis. Moreover, the QIA clinical kit results provide good contaminant retention, and it could be used as an alternative to specific extraction kits for matrices such as surfaces. In addition, according to Bio-Rad instruction for ddPCR data analysis, a sample is considered positive for a certain target when the analysis reveals at least three positive droplets. However, our study suggests that the cut-off values can be reduced, improving the sensitivity of the technique. Using the QIA method, the cut-off value can be reduced from three positive droplets to one for the N2 target and two for the N1 target (N1 = 1.5, N2 = 1.0). This represents an interesting outcome considering that a clinical ddPCR assay combined with clinical extraction methods was used for this study. Reducing the cut-off established by the manufacturer, confirms the high sensitivity of this method and demonstrates that combined with the QIA extraction method, ddPCR can return good results also on surface samples. Other interesting data were obtained from the ROC analysis performed to assess the accuracy of both extraction protocols through the number of copies of N1 and N2 obtained after the droplets’ reading process. The values reflected the differences between the two extraction methods. A careful observation of the previous data concerning ROC analysis shows that the cut-off value is considerably lower when referring to the N2 target obtained following the QIA extraction protocol (QIA: N1 = 9.665, N2 = 2.820; TRI: N1 = 32,430; N2 = 43.72). This supports the hypothesis that N2 target is more stable on surfaces and may be more suitable for SARS-CoV-2 detection on surfaces. Further analysis of a larger number of swab samples could confirm this hypothesis. If so, this could be the starting point for developing new environmental monitoring kits for SARS-CoV-2 detection.

When comparing ddPCR to other techniques, the differences between the two extraction methods stand out. Considering the comparison between RT-qPCR and QIA extraction ddPCR, ddPCR analysis showed a higher percentage of positive samples than RT-qPCR (QIA extraction ddPCR: 42/118; RT-qPCR: 27/118). The SE of 41% implies that QIA ddPCR has a moderate ability to detect positive samples compared to RT-qPCR, while the SP of 66% indicates a fair ability to correctly identify negative samples. The PPV of 26% for QIA ddPCR indicates that a portion of the positive samples detected is true positive, while the NPV of 79% shows a good ability to confirm negative samples. The percentage concordance of 60% and Cohen’s k of 0.1 for QIA ddPCR indicate a weak concordance between the two methods. For the comparison between RT-qPCR and TRI extraction ddPCR, a very low SE (7%) is observed, suggesting a poor ability to detect ddPCR-positive samples. However, the specificity 88% reveals a good ability to correctly identify negative samples. The PPV of 15% for TRI extraction ddPCR suggests that only a small fraction of the detected positive samples is true positive, while the NPV of 76% indicates a fair ability to confirm negative samples. The percent concordance of 69% and Cohen’s k of -0.1 for TRI ddPCR indicate fair percent concordance, but the negative Cohen’s k value suggests that concordance is worse than would be expected by chance.

Although a higher concordance rate has been recorded when comparing TRI ddPCR and RT-qPCR, TRI ddPCR shows limitations in terms of sensitivity and droplet generation, making it less practical as a primary screening method.

When comparing RT-LAMP to RT-qPCR, RT-LAMP detected a similar number of positives samples with respect to the reference method (RT-LAMP: 31/118; RT-qPCR: 27/118). The slightly high number of positive samples obtained with RT-LAMP may be explained by the differences between the methods compared. In fact, the amplification process of RT-LAMP is different from that of RT-PCR in several aspects: i) the number of primers used in RT-LAMP is greater than in RT-PCR. This makes RT-LAMP a more specific method as confirmed by the comparison of the two methods performed. Moreover RT-LAMP is the only assay developed for surfaces employed in this study, based on N and S genes target, respecting the target used by RT-PCR assay (RdRp gene). The use of different target could explain the different levels of detection observed. The results obtained showed that RT-LAMP has a relatively low SE (30%). This suggests that RT-LAMP could detect several false negatives compared with RT-qPCR. Having a SP of 75% implies that although RT-LAMP can better recognize negative samples, false positives may be detected. In terms of predictive value, a PPV of 26% suggests that many of the positive samples detected by LAMP may be false positives, while NPV of 78% indicates that LAMP is quite effective in detecting negative samples. The low Cohen’s k value (0.0414) and the percentage of concordant sample (64%) further support the slight agreement between the two methods. These results indicate that while RT-LAMP is useful for initial screening because of its rapidity and ease of use, positive results must be confirmed with a more reliable method such as RT-qPCR to ensure accurate identification of SARS-CoV-2 on surfaces. The differences between the two extraction methods used in ddPCR previously described also affect the comparison between ddPCR and RT-LAMP. RT-LAMP detected less positives respect to QIA extraction ddPCR but fewer than TRI extraction ddPCR (RT-LAMP 31/118; QIA extraction ddPCR: 42/118, TRI extraction ddPCR: 13/118). The SE of RT-LAMP obtained from the comparison with TRI extraction ddPCR was extremely low (8%), indicating that the method may not be effective in detecting positive samples. On the contrary, the SP of 71% suggests that RT-LAMP is reliable in detecting negative samples, reducing false positives. The PPV of 3% indicates that only a small portion of the positive samples detected by ddPCR are true positives, while the NPV of 86% suggests that the method is quite effective in confirming negative samples. RT-LAMP results show 64% concordance and a Cohen’s k of -0.13. These values suggest a discordance between the results obtained with RT-LAMP and ddPCR. Comparing RT-LAMP with QIA extraction ddPCR, the SE of 26% obtained points the moderate ability of RT-LAMP to detect positive samples compared to ddPCR, while a SP of 74% confirm the fair ability of RT-LAMP to correctly identify negative samples, although there are several false positives. A PPV of 35% indicates that only a portion of the positive samples detected by ddPCR is true positive, while the VPN of 64% suggests that the method is quite effective in confirming negative samples. The percentage concordance of 57% and a Cohen’s k coefficient of 0.01 indicate poor concordance between the results obtained by RT-LAMP and ddPCR.

Apparently, the results obtained from the comparison between ddPCR and RT-LAMP show a better performance of the RT-LAMP technique when compared with TRI extraction ddPCR, oppositely to the comparison with QIA extraction ddPCR because of more concordant samples (TRI extraction ddPCR: 76/118 vs QIA extraction ddPCR: 67/118) and for a higher NPV (TRI extraction ddPCR: 86% vs QIA extraction ddPCR: 64%). However, these data need to be reevaluated considering the comparison between the two extraction methods previously discussed and, considering that TRI extraction ddPCR detects a lower number of positive samples and showed more difficulties in sample partitioning when compared to the QIA extraction ddPCR method.

Despite these remarkable results, ddPCR and RT-LAMP showed the same limitations. For example, as other molecular biology techniques, ddPCR and RT-LAMP do not ensure that a positive result is due to a viable and infectious pathogen. In addition, suboptimal SE and SP values were observed compared with the reference method. The use of clinical kits instead of specifically surface-designed kits could explain these discrepancies: surfaces might contain substances able to interfere with the RNA extraction or amplification. Other causes may include the use of kits designed for different targets detection and the nature of the techniques being compared. Finally, the prevalence of the disease within the population may have been a relevant factor since, over the course of the sampling campaign, it was not constant.

In consideration of these critical findings, although the RT-LAMP method offers a quick and simple solution, its moderate sensitivity and specificity compared to RT-qPCR suggests the need for improvements for more accurate detection. However, considering that RT-LAMP is a qualitative and handling assay that does not require special instrumentation for the analysis delivering results within 40 minutes, it is suitable for screening in community settings like schools, workplaces, and healthcare facilities in preventive approach as well as during outbreaks.

RT-LAMP could be used effectively to identify true negative surfaces to assess the hygienic status of the facilities and the effectiveness of surface sanitation processes. It could be useful, for example, before allowing access to a sanitized area following cases or outbreak.

## Conclusions

The SARS-CoV-2 outbreak demonstrated that the course of a pandemic is unpredictable, and containment efforts are extremely challenging. Environmental monitoring has proven crucial in detecting changes in pathogens in the environment, potentially signaling the emergence of new dangerous diseases before their worldwide spread. In this scenario, this study proposed an environmental monitoring model that could be adapted not only to the pathogen of interest, in this case SARS-CoV-2, but also to others linked to human health. In line with the interdisciplinary One Health approach, this study aims to preserve the public health safety studying the behavior of SARS-CoV-2 virus on “unconventional” pathways as fomites, integrating the clinical knowledge of the pathogen with its prevalence in environment. The approach proposed could be applied in low-risk communities such as schools, workplace and recreational environments, as well as in high-risk settings, like hospital wards frequented by vulnerable patients (e.g. oncological, immuno-compromised, elderly, etc.). Especially in intensive care units, as well as in operating rooms, oncology, infectious disease and long-term care wards, a tool such as RT-LAMP represents a fast and cost-effective way to monitor the presence of SARS-CoV-2 and other pathogens. In addition, in healthcare settings, constantly subjected to sanitization processes and with high level of disinfection required, RT-LAMP can be used to assess the hygienic conditions of the environment and to confirm (or not) the effectiveness of sanitization processes, often carried out by external suppliers. Considering the overall properties of each technique, the results of this study suggest the use of RT-LAMP as a useful method to screen negative from positive surfaces and, in case of a positive outcome, if possible, using a sensitive quantitative test such as ddPCR to confirm the result. As an alternative to ddPCR, it would be appropriate to use RT-qPCR as a confirming test, a widespread technology in the laboratory routine, but using the kit for appropriate matrices. At moment several RT-LAMP assays are commercially available, but for further cost reduction, it is possible to design and customize the assay and work with cheap and available laboratory equipment such as a thermal block or thermostat bath. Therefore, this study is an inspiration for the development of new protocols able to support the environmental surveillance guidelines for pathogens of clinical interest.

In conclusion, this study emphasizes a proactive and preventive approach to emerging pathogens and proposes innovative strategies to achieve this purpose by comparing different molecular biology techniques. It could represent the first step to assess guidelines for an environmental monitoring of SARS-CoV-2 even if the air investigation is also required, considering that SARS-CoV-2 is an airborne pathogen. Only using an integrated approach, the environmental and clinical pathogens spread prevention will be possible.
